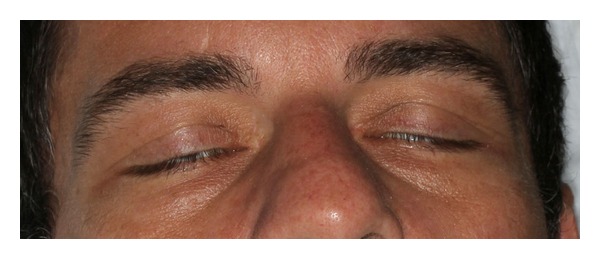# Presentation of Bilateral Peripheral Seventh Cranial Nerve Palsy in an HIV Patient

**DOI:** 10.1155/2012/267405

**Published:** 2012-08-05

**Authors:** Lisa M. Ruiz, Batool Kirmani

**Affiliations:** ^1^Department of Psychiatry, Scott & White Healthcare, Temple, TX 76508, USA; ^2^Department of Neurology, The Neuroscience Institute, Scott & White Healthcare, Texas A&M Health Science Center College of Medicine, Temple, TX 76508, USA

## Abstract

Neurological manifestations in patients infected with human immunodeficiency virus can significantly increase overall morbidity and mortality. These complications are neither limited to a specific location in the nervous system nor a focal time period in the disease's progression. A literature review yielded several cases of peripheral facial palsy associated with HIV seropositivity, but few cases have been reported where the patient had bilateral peripheral facial palsy. In this paper, we present a patient with bilateral peripheral facial palsy and aseptic meningitis in the context of newly diagnosed HIV.

## 1. Introduction

Human immunodeficiency virus (HIV) is a retrovirus that affects the immune system by targeting T cells with CD4 receptors. Since its identification, the medical community has become increasingly knowledgeable regarding the many opportunistic infections that can affect these immunosuppressed individuals. Unfortunately, neurological manifestations associated with HIV seropositivity are also increasing the morbidity and mortality rates of these patients. The virus can affect the nervous system at any level producing a variety of clinical presentations [1]. Some complications include neuritis, myelitis, aseptic meningitis, neuropathy, myelopathy, encephalopathy, and cognitive alterations [1, 2]. Many of these complications occur within the first three weeks of infection, while others do not manifest until late in the disease process such as encephalopathy and dementia [2]. 

This paper presents a patient with early stage neurological complications, bilateral peripheral facial palsy, and aseptic meningitis, in the context of newly diagnosed HIV. 

## 2. Case Report

 A 41-year-old homosexual male presented for bitemporal headache, inability to close his right eye, perioral numbness, and myalgias. In the prior two weeks, he had extensive diagnostic workup for abdominal pain. He was subsequently diagnosed with viral hepatitis and HIV, confirmed with western blot. He had tested negative for HIV one year before. Two days after hospital discharge he developed a bitemporal headache that originated posteriorly and was not relieved with Vicodin. He also complained about difficulties closing his eyes, right worse than left, and inability to close his mouth completely causing food to dribble out of his mouth. He denied any visual changes or difficulty swallowing. He denied ever having either of these complaints previously. He had not travelled outside of the United States and knew of no illnesses among his personal contacts. He has had three partners in the past year with his last sexual encounter six weeks ago. He denied any intravenous drug use, is a nonsmoker, and drinks socially once a month. Review of systems also revealed mild photophobia, shoulder pain, cough, periorbital paresthesias, night sweats, and nausea otherwise, negative for fevers, weight loss, dyspnea, and chest pain.

**Figure 1:**
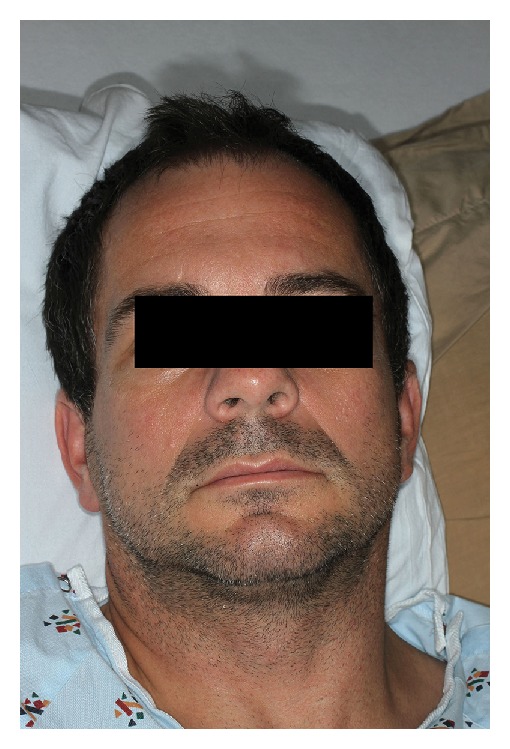


**Figure 2:**
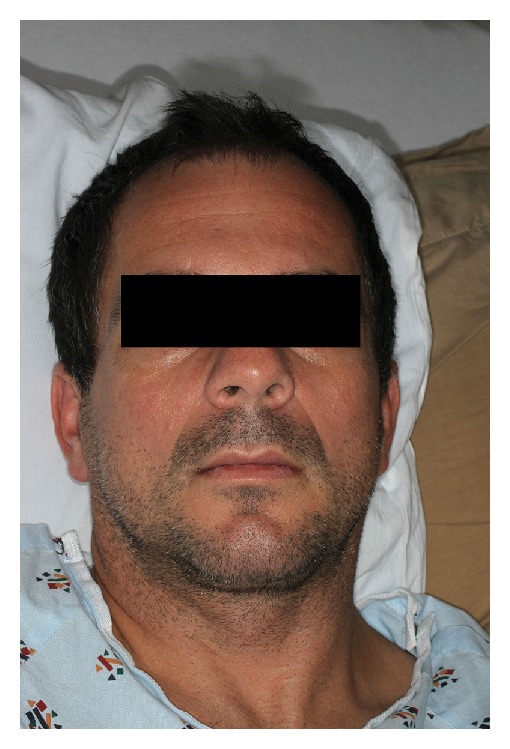


At admission, his vitals were stable. Objectively, the patient appeared to be quite anxious, shaking intermittently. His neurological exam was significant for an inability to smile, frown, close his eyes completely, or puff out his cheeks (Figures 1, 2, and 3). His exam was otherwise unremarkable.

At admission, his CBC & CMP were unremarkable. A lumbar puncture was done and the cerebrospinal fluid (CSF) contained protein of 87.6, glucose of 47, WBC of 41, lymphocytes of 98%, monocytes of 2%, and gram stain and cultures showed no organisms. CSF analysis was negative for *Cryptococcus* Ag, CMV, VDRL, HSV, and arbovirus IgG and IgM. His serum was negative for parvovirus IgG and IgM, CMV, EBV, HSV, Enterovirus, arbovirus IgG and IgM, *Histoplasma* Ag, *Coccidiodes* Ab. VDRL and FTA-ABS antibodies were nonreactive. Lyme antibodies IgM and IgG were negative. California encephalitis virus, St. Louis encephalitis virus, eastern equine encephalitis virus, and West Nile virus were all negative for IgG and IgM, respectively. From his previous admission, his viral load was log 6.11, 1290000 copies/mL and CD4 count was 345. 

His CT of the head and MRI of the brain without contrast showed no acute intracranial abnormalities.

He was ultimately diagnosed and treated for lymphocytic predominant meningitis with bilateral peripheral facial neuropathy secondary to HIV. He was given supportive care for the meningitis and neuropathy, started on Atripla for HIV infection and referred to the infectious disease clinic for followup on an outpatient basis. 

## 3. Discussion

This patient's bilateral peripheral facial palsy and aseptic meningitis can be attributed to his recently diagnosed HIV seroconversion. The extensive diagnostic work-up did not reveal another infectious agent that could have contributed to his neurological complications. This is important as thorough histories along with adequate laboratory tests are critical in determining a possible etiology for bilateral diplegia [3]. 

Bilateral peripheral facial palsy with recent HIV seroconversion has been previously reported in few other case reports and is considered to be a rare occurrence [4]. This lack of reporting is not surprising since bilateral facial palsy only accounts for 0.3–2% of cases and is reported to have an incidence of 1 in 5 million inhabitants per year [5]. 

Kim et al. reported a similar case of bilateral peripheral facial palsy and aseptic meningitis in a patient with HIV [4]. However, the patients differ in their presentations relative to their HIV diagnosis. Their patient presented with bilateral peripheral facial palsy and was eventually diagnosed with HIV. Our patient had been diagnosed with HIV one week prior to the manifestation of his neurological complication. In further comparison, both patients had normal MRI results as opposed to increased signal enhancement of the facial nerves. Sattoretti-Schefer et al. noted that intraneural edema or neural fiber swelling may be present due to viral localization that would cause this MRI abnormality [6]. Both patients were treated for their lymphocytic predominant meningitis and started on HIV medication prior to hospital discharge; however, neither had any improvement of their HIV neuropathy. 

Fortunately for these two patients, their neurological manifestations were quickly associated with HIV, and, consequently, the underlying infection could be treated. A review article on facial palsies encourages physicians to consider more exotic causes before labeling such presentations as Bell's palsy, which is a diagnosis of exclusion and an idiopathic condition [7]. This is not unreasonable as facial palsy may become a more common presentation due to the increasing incidence of HIV, especially among younger age groups [8]. High suspicion may facilitate earlier diagnosis of HIV infection leading to earlier treatment and possibly decreased dissemination.

## 4. Conclusion

Bilateral peripheral facial palsy is currently a rare manifestation, especially in the context of HIV infection.

## Figures and Tables

**Figure 3:**